# Clinically Deployable Bioelectronic Sensing Platform for Ultrasensitive Detection of Transferrin in Serum Sample

**DOI:** 10.3390/bios13030406

**Published:** 2023-03-20

**Authors:** Harleen Kaur, Prasanthi Chittineedi, Ravi Shankar Bellala, Venkata Madhavi Bellala, Sandeep Singh, Rohini Kumari, Pranjal Chandra, Santhi Latha Pandrangi, Surinder P. Singh

**Affiliations:** 1 CSIR—National Physical Laboratory, Dr. K. S. Krishnan Marg, New Delhi 110012, India; 2 Onco-Stem Cell Research Laboratory, Department of Biochemistry and Bioinformatics, GITAM School of Science, GITAM (Deemed to be) University, Visakhapatnam 530045, India; 3 Department of Clinical Oncology, Omega Hospitals, Visakhapatnam 530040, India; 4 Department of Pathology, GITAM Institute of Medical Sciences and Research, Visakhapatnam 530045, India; 5 Laboratory of Bio-Physio Sensors and Nanobioengineering, School of Biochemical Engineering, Indian Institute of Technology (BHU), Varanasi 221005, India; 6 Academy of Scientific and Innovative Research (AcSIR), CSIR-HRDC Campus, Ghaziabad 201002, India

**Keywords:** ferritin, immunosensors, reduced graphene oxide, transferrin, electrochemical detection

## Abstract

Varying levels of transferrin (Tf) have been associated with different disease conditions and are known to play a crucial role in various malignancies. Regular monitoring of the variations in Tf levels can be useful for managing related diseases, especially for the prognosis of certain cancers. We fabricated an immunosensor based on graphene oxide (GO) nanosheets to indirectly detect Tf levels in cancer patients. The GO nanosheets were deposited onto an indium tin oxide (ITO)-coated glass substrate and annealed at 120 °C to obtain reduced GO (rGO) films, followed by the immobilization of an antibody, anti-Tf. The materials and sensor probe used were systematically characterized by UV–Visible spectroscopy (UV–Vis), X-ray diffraction (XRD), atomic force microscopy (AFM), and Fourier transform infrared spectroscopy (FTIR). Cyclic voltammetry (CV), electrochemical impedance spectroscopy (EIS), and differential pulse voltammetry (DPV) were also used for the stepwise sensor probe characterizations and Tf detection in serum samples, respectively. The anti-Tf/rGO/ITO immunosensor DPV output demonstrated an excellent Tf detection capability in the linear range of 0.1 mg mL^−1^ to 12 mg mL^−1^ compared to the enzyme-linked immunosorbent assay (ELISA) detection range, with a limit of detection (LOD) of 0.010 ± 0.007 mg mL^−1^. Furthermore, the results of the fabricated immunosensor were compared with those of the ELISA and autobioanalyzer techniques, showing an outstanding match with < 5% error and demonstrating the immunosensor’s clinical potential.

## 1. Introduction

Cancer is the leading cause of mortality worldwide, and the American Cancer Society was expecting 1,918,030 new cancer cases and 609,360 fatalities in 2022 [1,2]. Despite many technological advancements in cancer care, the 5-year survival rate of patients with many types of cancers is not encouraging. Residual cells after surgery or other therapies can lead to tumor recurrence with increased aggressiveness and metastasis. These residual cells are considered causative agents for therapeutic resistance and possess stem cell-like properties; therefore, they are called cancer stem cells (CSCs) [3]. The CSCs, having a self-renewing character, are found in almost all types of cancers and contribute to tumor expansion, recurrence, and metastasis after therapy. Malignant tumor cells need more resources than healthy cells in order to maintain their hyperproliferative nature and survive in the natural tumor microenvironment [4]. This implies that the nutrients required for cancer metabolism can serve as biomarkers to identify various cancers. According to recent studies, anemia is one of the primary risk factors for the development of cancer. Anaemia is an iron deficiency disorder characterized by low hemoglobin or high ferritin levels [5]. It is a well-demonstrated fact that cancer cells and CSCs store bulk iron in the form of ferritin, making these cells more sustainable in the harsh tumor microenvironment [6]. The body’s iron stores are mainly regulated by hepcidin, ferroportin, and Tf, which act as iron transporters [7]. Hepcidin and ferroportin are confined to the liver and play a vital role in exporting absorbed iron to various parts of the body. At the same time, Tf is omnipresent in all cell types, shows a high affinity to ferritin, and delivers iron through receptor-mediated endocytosis. Cancer cells and CSCs need bulk ferritin to survive and proliferate in hypoxic conditions. However, serum Tf undergoes saturation under high ferritin levels, leading to low Tf levels in serum [8]. It has been observed that with an increase in tumor staging and grading, there would be an enhanced rate of iron metabolism, suggesting that as the tumor grade increases, ferritin levels increase. In contrast, Tf levels drastically decrease, indicating that serum Tf may serve as a biomarker for detecting cancer stages and prognosis [9,10,11,12]. The variation in Tf levels is also associated with other diseases such as hepatitis, rheumatism, cirrhosis, malignant tumor, leukemia, and nephritic syndrome, suggesting the importance of its accurate and rapid detection [13]. Interestingly, a population-based study by Ellervik C et al. showed that cancer risk can be determined by the hemochromatosis genotype and transferrin saturation (TS). The results of their study revealed that high TS in women and the hemochromatosis genotype in men could be linked to an elevated cancer risk [14]. Iron transfer requires a high concentration of Tf; however, low Tf levels are found in TS situations. Therefore, we hypothesize that low levels of Tf in the bloodstream can be used as a diagnostic marker to detect cancer at an early stage.

The ELISA, histopathological assays such as estimates of TIBC and ferritin, immunochemical nephelometry, radioimmunoassay, capillary zone electrophoresis, and other techniques are frequently employed in the detection of Tf and other biomarkers [15]. Although these methods are accurate and adequately sensitive, they take a long time and require specific laboratory conditions, as well as trained staff, making the tests more cumbersome [16,17,18]. The high sensitivity, quick detection, wide dynamic range, and affordable price of electrochemical sensors make them a desirable alternative for detection [19,20,21]. In recent years, many researchers have employed electrochemical methods to detect different biomarkers. For instance, Rocha et al. constructed an impedimetric immunosensor using rGO to detect staphylococcal enterotoxin A in milk samples [22]. Wang et al. developed an electrochemical nanosensor based on a nanocomposite of rGO, polyethyleneimine, silver nanoparticles, and nafion to detect arsanilic acid (ASA) [23]. Braz et al. used a graphene–solid binding peptide to detect SARS-CoV 2 antibodies [24]. Ketmen et al. fabricated GO–magnetic nanoparticles on polystyrene–polydopamine electrospun nanofibers for the electrochemical sensing of C-reactive protein (CRP) [25]. Tajik et al. fabricated an electrochemical immunosensor using ZnFe_2_O_4_/rGO to detect hydrazine [26]. Lastly, Gu et al. used doped nitrogen–rGO@carboxylated MWCNTs/chitosan@gold nanoparticles to identify CA125 in serum via the electrochemical route [27].

Similar attempts have been made by researchers to develop a quick, easy, and affordable way for the point-of-care detection of Tf biomarkers. A label-free surface plasmon resonance biosensor for Tf detection based on GO and gold nanorods was developed by Zhang et al., showing dynamic linearity in the Tf concentration range of 0.375–40.00 µg/mL [28]. Matisiak-Brynda et al. designed a unique immunosensor based on magnetic nanoparticles for the direct sensing of Tf. Using a covalent bond with a cysteamine monolayer, the carbon-encapsulated magnetic nanoparticles were initially anchored to the gold surface, which was used for antibody immobilization. Hence, direct electron transfer between the protein and the electrode surface was achieved through the integration of magnetic nanoparticles with monoclonal antibodies, without any need for a mediator molecule. The results showed linearity in the range of 5 × 10^−7^ and 5 × 10^−2^ g dL^−1^, with a limit of detection of 24.0 ± 5.2 ng/dL [29]. Another label-free immunosensor was designed by Kong et al. by utilizing a disposable carbon electrode modified with luminol-reduced gold nanoparticles (Lu-Re-GNP) to detect Tf in serum [30]. The stable and potent ECL emission of the Lu-Re-GNP-modified sensing platform can be used to identify the target antigen with the aid of the unconjugated antibody bound to the electrode. The immuno-binding event caused by TRF resulted in a reduction in the ECL intensity. This sensing platform is specific and stable with a wide linear range of 0.10–18 ng mL^−1^ and a detection limit of 0.033 ng mL^−1^. Miao et al. constructed a highly selective MIP-based labelled phosphorescent sensor to detect Tf. In their investigation, room-temperature phosphorescent Mn-ZnS QDs (RTPQDs), which have a good anti-interference capability against background fluorescence, were chosen as the luminous materials and mesoporous nano-SiO2 was employed as a substrate, which enabled the development of more three-dimensional fine-recognition sites. Hence, RTP protein mesoporous-imprinted microspheres (SiO_2_–PQDs–MIPs) were used to detect Tf, with linearity in the range of 0.05–1.0 μM and a LOD of 0.014 µM [31]. Zhang et al. constructed SERS nanotags labeled with Fe_3_O_4_@Au@cyclodextrin-molecularly imprinted polymer for ultrasensitive detection of Tf [32]. In order to specifically focus on Tf, magnetic MIP (MMIP) was designed using *β*-CD as the functional monomer. The calibration curve showed linearity in the range of 0.1–10^5^ ng/mL with a LOD of 100 pg/mL. The above-discussed labeled and label-free electrochemical biosensors have shown promising results for the detection of Tf biomarkers. However, to the best of our knowledge, no biosensing investigations for Tf detection have been carried out that correlate the variation of Tf in context to cancer patients and validated with clinical gold standard techniques parallely.

Nanobiosensors have shown improved analytical performance due to their large surface area and increased catalytic and conductive activity [33,34,35,36]. However, most of them still suffer from drawbacks such as material degradation, bioreceptor stability, and sluggish electron kinetics. Recently, GO and rGO have attracted increasing interest in their biosensing applications owing to their 2D sheet-like planer structure, good conductivity, and oxygen functionalities that facilitate the easy immobilization of biomolecules [37,38,39,40]. GO is produced by oxidizing and exfoliating layers of graphite that have a significant number of oxygen-containing molecules, such as hydroxyl, carboxyl, or epoxy groups on their carbon edges and basal plane. rGO is produced by significantly reducing the quantity of oxygen-containing groups in GO through chemical or thermal reduction. When the C/O ratio increases, the structure and properties of rGO become similar to those of pristine graphene. In addition, the enhanced electron transfer facilitated by the π network of carbon atoms in rGO makes it an ideal candidate for electrode fabrication for immunosensors. Many researchers have exploited the intriguing properties of GO and rGO by attaching numerous nanomaterials and biomolecules to detect various clinically relevant molecules with high sensitivity [41,42,43,44].

The present study focuses on developing a cost-effective, rGO-based immunosensor to detect Tf in serum samples of cancer patients. The immunosensor is thoroughly characterized and validated by UV–Vis, XRD, AFM, FTIR, CV, EIS, and DPV. The results of the Tf analysis of the blood samples using the fabricated immunosensor are compared with those of standard techniques such as ELISA and an autobioanalyzer in a laboratory setting. It is observed that the results obtained using the immunosensor are in excellent agreement with the results of the standard methods, with < 5% variation. The immunosensor is tested for its selectivity against various interfering molecules and the long-term stability is also evaluated to establish its shelf-life.

## 2. Materials and Methods

### 2.1. Chemicals and Materials

Graphite flakes (Bay Carbon), hydrogen peroxide (H_2_O_2_), sodium chloride (NaCl), potassium permanganate (KMnO_4_), sodium nitrate (NaNO_3_), and potassium chloride (KCl) were purchased from Merck, Germany. Potassium phosphate monobasic (KH_2_PO_4_), sodium phosphate dibasic dihydrate (Na_2_HPO_4_), ethanolamine (HOCH_2_CH_2_NH_2_), potassium hexacyanoferrate (II) trihydrate (K_4_Fe(CN)_6_·3H_2_O), potassium hexacyanoferrate (III) (K_3_Fe(CN)_6_), N-(3-dimethyl aminopropyl)-N’-ethyl carbodiimide hydrochloride (EDC), sulfuric acid (H_2_SO_4_), and N-hydroxysuccinimide (NHS) were procured from Sigma Aldrich, USA. Glass substrates with an ITO coating on one side (glass thickness: 1.1 mm; resistivity: 15–20 cm) were bought from Macwin in India. Tf antibody and anti-rabbit IgG peroxidase-labeled secondary antibody were procured from Thermo Scientific. Human holo-transferrin protein was procured from Sigma-Aldrich. All the reagents were synthesized in milli-Q water, with a resistivity of 18 M Ω cm. 

### 2.2. Serum Sample Collection

All clinical studies and sample collections were performed at GITAM Institute of Medical Science and Research (GIMSR) and Omega Hospitals, Visakhapatnam, India. Ethical Clearance for this experiment was obtained from the Institutional Ethical Clearance Committee (IEC) of both GITAM Institute of Medical Science and Research (GIMSR) (GIMSR/Admn./Ethics/approval/IEC-3/2021, dated 13 August 2021) and Omega Hospitals, Visakhapatnam, India (IEC-OMEGA, dated 27 August 2021). Ten blood serum samples from cancer patients at Omega Hospitals OPD from August 2021–February 2022 were collected after obtaining the patients’ signatures on the informed consent form. Red serum vacutainers (BD Bioscience) were used to collect a 5 mL blood sample from each patient. The collected blood samples were immediately centrifuged at 1000 g for 10 min and serum was collected and stored at −80 °C in 1.5 mL Eppendorf tubes until further analysis.

### 2.3. Synthesis of Graphene Oxide

The GO nanosheets were prepared utilizing a modified Hummer’s method [45], where 0.5 g of NaNO_3_ and 0.5 g of graphite powder were added to a conical flask and stirred in 23 mL of H_2_SO_4_ (98%) for 1 h at 25 °C. The solution was then transferred to an ice bath (4 °C) and finely crushed KMnO_4_ (1.5 g) was added to it slowly under vigorous stirring for 1 h. The solution was then brought to 40 °C and stirring continued until a dark-green slurry was obtained. The reaction mixture turned yellow brown when 50 mL of ice-cold deionized (DI) water was slowly added. Furthermore, the solution was sonicated for 15 min, followed by the addition of 2 mL of H_2_O_2_ (30%) and stirring for another 15 min to terminate intermediate reactions. After that, the obtained solution was rinsed multiple times with DI water until the pH became neutral. Lastly, the solution was lyophilized to obtain dry GO nanosheets.

### 2.4. Characterization Techniques

The synthesis of GO was confirmed using structural, optical, and morphological characterizations. X-ray diffraction (XRD) was performed to validate the crystalline structure using a Rigaku Miniflex 600 diffractometer, Tokyo, Japan, with CuKα radiations in the 2θ value range of 5° to 80°. FTIR spectroscopy was performed using a Nicolet iS50, ThermoFisher Scientific, Waltham, Massachusetts, USA, in the wavenumber range of 400 cm^−1^ to 4000 cm^−1^, to identify the GO nanosheets’ functional groups and surface functionalizations. The optical studies of the GO were carried out using an ultraviolet-visible NIR spectrophotometer (Cary 5000, Agilent Technologies, Santa Clara, California, USA) in a wavelength range of 200 nm to 800 nm. The morphological studies were conducted using scanning electron microscopy (SEM) with a machine by Tescan Analytics, Fuveau, France. AFM images were captured using an NS50 Veeco Instrument in tapping mode (New York, NY, USA). The electrode fabrication was performed using a spin coater (WS-650MZ-23NPP, Laurel Technologies Corporation, North Wales, Pennsylvania, USA) at 3000 rpm. The electrochemical studies were conducted on a compact, portable device at RT on a PalmSens 3 (Palm Instruments BV, Houten, The Netherlands) using a three-electrode system consisting of Ag/AgCl as a reference electrode dipped in 0.1 M KCl solution, Cu as a working electrode, and Pt as a counter electrode.

### 2.5. Fabrication of rGO/ITO Electrode

The lyophilized GO was dispersed in DI water using ultra-sonication for 10 min to obtain a 0.5 mg/mL aqueous dispersion of GO. The prepared solution was spin-coated onto an ITO-coated glass substrate. Before deposition, the ITO electrodes were thoroughly cleaned with detergent, DI water, isopropyl alcohol, and acetone. After deposition, the fabricated GO films were reduced thermally by annealing them at 120 °C for 4 h in a box furnace to obtain rGO films. The rGO/ITOs films were characterized using electrochemical techniques, CV at varying scan rates ranging from 0.01 Vs^−1^ to 0.15 Vs^−1^ in 20 mL of 3 mM Zobell’s solution.

### 2.6. Fabrication of Anti-Tf/rGO/ITO Immunosensor

**Scheme 1:**
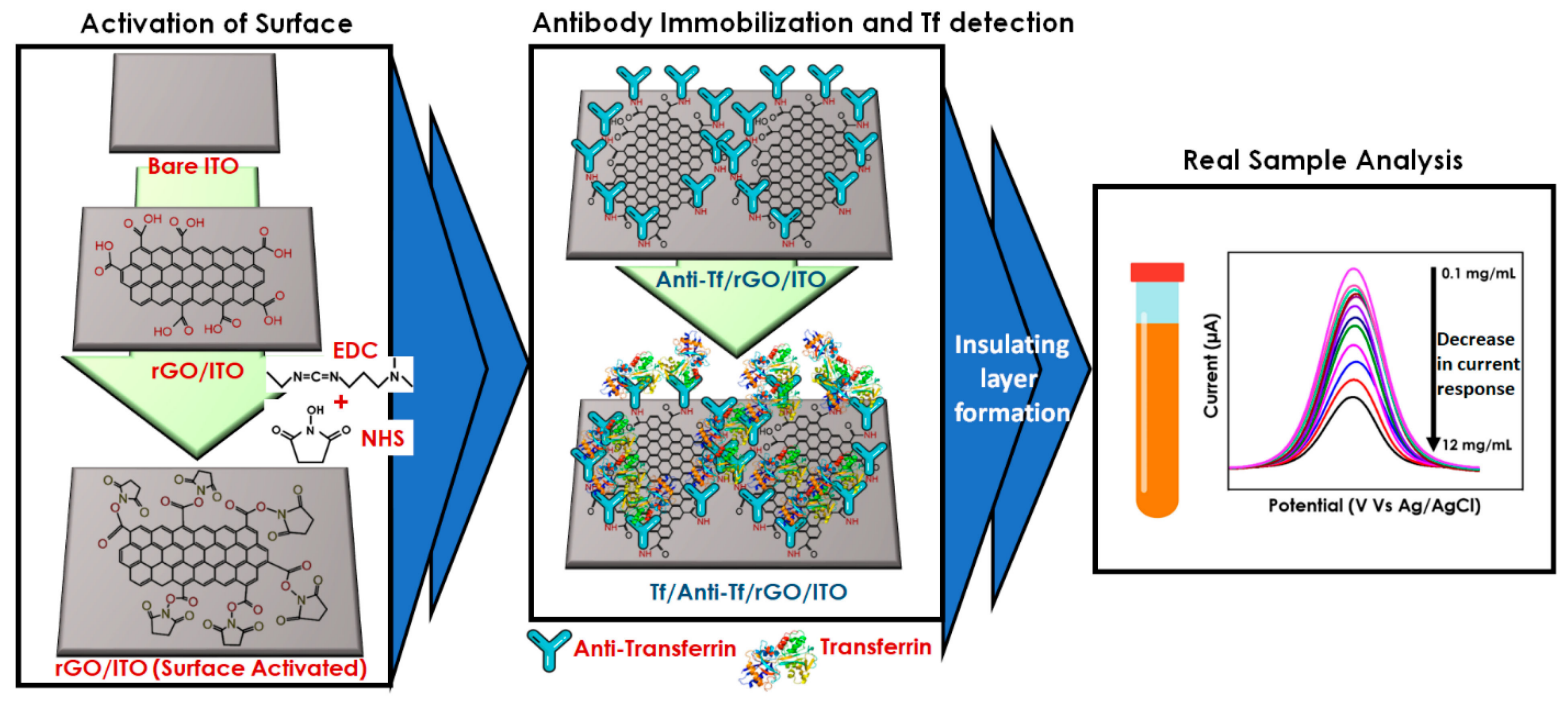
Fabrication and Tf detection strategy using anti-Tf/rGO/ITO immunosensor.

Prior to Tf functionalization, the rGO/ITO surface was subjected to 60 µL of 0.5 M EDC and 0.1 M NHS mixed in a 1: 1 (*v*/*v*) ratio to activate the -COOH functional groups present on the rGO surface. After incubation for 1 h, the electrodes were rinsed with DI water. Thereafter, 20 µL of 1 µg/mL of anti-Tf was dropcasted onto the activated rGO/ITO films, kept for 4 h, and then washed with phosphate buffer saline (PBS) solution (10 mM, pH: 7.4) to eliminate unbound antibodies. Furthermore, the active sites on the antibody-coated electrode were blocked by 20 µL of 1% ethanolamine for 20 min and washed with PBS. Finally, these electrodes were characterized using CV at a scan rate of 0.05 Vs^−1^ in 20 mL of 3 mM Zobell’s solution and by EIS. The stepwise fabrication of the anti-Tf/rGO/ITO bioelectrode and the detection of Tf is shown in Scheme 1. The underlying principle behind the detection is that after interacting with the anchored antibody, Tf produces insulation on the electrode surface. Higher Tf concentrations result in an increase in the formation of Tf–antibody conjugates, which increases the insulation of the electrode surface and restricts the transfer of electrons, consequently affecting the analytical signals.

### 2.7. Transferrin Detection Using Anti-Tf/rGO/ITO Electrode

Different concentrations of Tf antigen ranging from 0.1 mg mL^−1^ to 12 mg mL^−1^ were synthesized in PBS solution. The fabricated anti-Tf/rGO/ITO electrodes were incubated with 20 µL of each concentration of Tf antigen for 15 min and then washed with PBS buffer to eliminate excess antigens. The immunoreactions on the electrodes were analyzed using the DPV technique at a 0.02 Vs^−1^ scan rate, with a pulse time of 0.07 s in Zobell’s solution. Furthermore, the anti-Tf/rGO/ITO electrodes were tested on real samples obtained from different cancer patients. The obtained serum from cancer patients was used to investigate Tf by incubating 20 µL of unknown serum samples on the fabricated immunosensor for 30 min. Excess serum was washed with PBS solution, the DPV response was recorded, and the results were compared with those of the ELISA and autobioanalyzer techniques.

### 2.8. Specificity Studies on Anti-Tf/rGO/ITO Immunosensor

The fabricated immunosensor was exposed to different antigens such as S-100, IL-8, h-TERT, Tf, and a mixture (containing 5 µL of each of the aforementioned analytes) using 20 µL of 2 mg mL^−1^ prepared in PBS buffer_._ These antigens were deposited individually on the anti-Tf/rGO/ITO electrode for 30 min and later washed with PBS to remove excess protein. The selectivity was determined by performing DPV studies in Zobell’s solution. 

### 2.9. Estimation of Transferrin Levels through ELISA 

To quantify the amount of Tf present in the blood serum of cancer patients, ELISA was performed. Tf primary antibody was applied to the 96-well plates and incubated at 40 °C overnight. After incubation, the antibody was retrieved and the plate was rinsed with wash buffer (1 × TBST) twice. An amount of 100 µL of 5% BSA was added to the plate to block unspecific interactions and it was then incubated on an orbital shaker for 30 min, followed by the addition of 100 µL of a known concentration of protein standards and undiluted serum samples. The plate was further incubated on an orbital shaker at room temperature for 2 h and then washed twice, followed by incubating it with a secondary antibody for 2 h at room temperature. After subsequent washing, the plate was incubated with a chromogenic substrate, 2,2’-azino-bis (3-ethylbenzothiazoline-6-sulfonic acid (ABTS), and incubated for 1 h at room temperature. Stop buffer (0.5 M NaOH) was used to stop the enzyme–substrate interaction, and a microplate reader (BO RAD PR 4100) was used to record the samples’ optical densities (OD) at 450 nm.

### 2.10. Validation of Serum Transferrin Levels through Autobioanalyzer

The ELISA results were further validated using an autobioanalyzer. Briefly, serum ferritin levels from the patients were measured using a COBAS C311 analyzer as per the manufacturer’s instructions. Tf-bound ferritin was treated with ascorbic acid, resulting in ferritin release from Tf. The released ferritin, which was present in a ferric ion state, was reduced to a ferrous ion state and then quantified using a ferrozine colorimetric reaction. The concentration of ferritin was directly proportional to the color intensity. The obtained ferritin levels were further used to calculate the Tf levels using Equation (1) [46]:

(1) $$Transferrin\;\left(mg/dL\right)=iron\;\left(µg/dL\right)/1.4$$

## 3. Results and Discussion

### 3.1. Physical Characterization of GO

**Figure 1:**
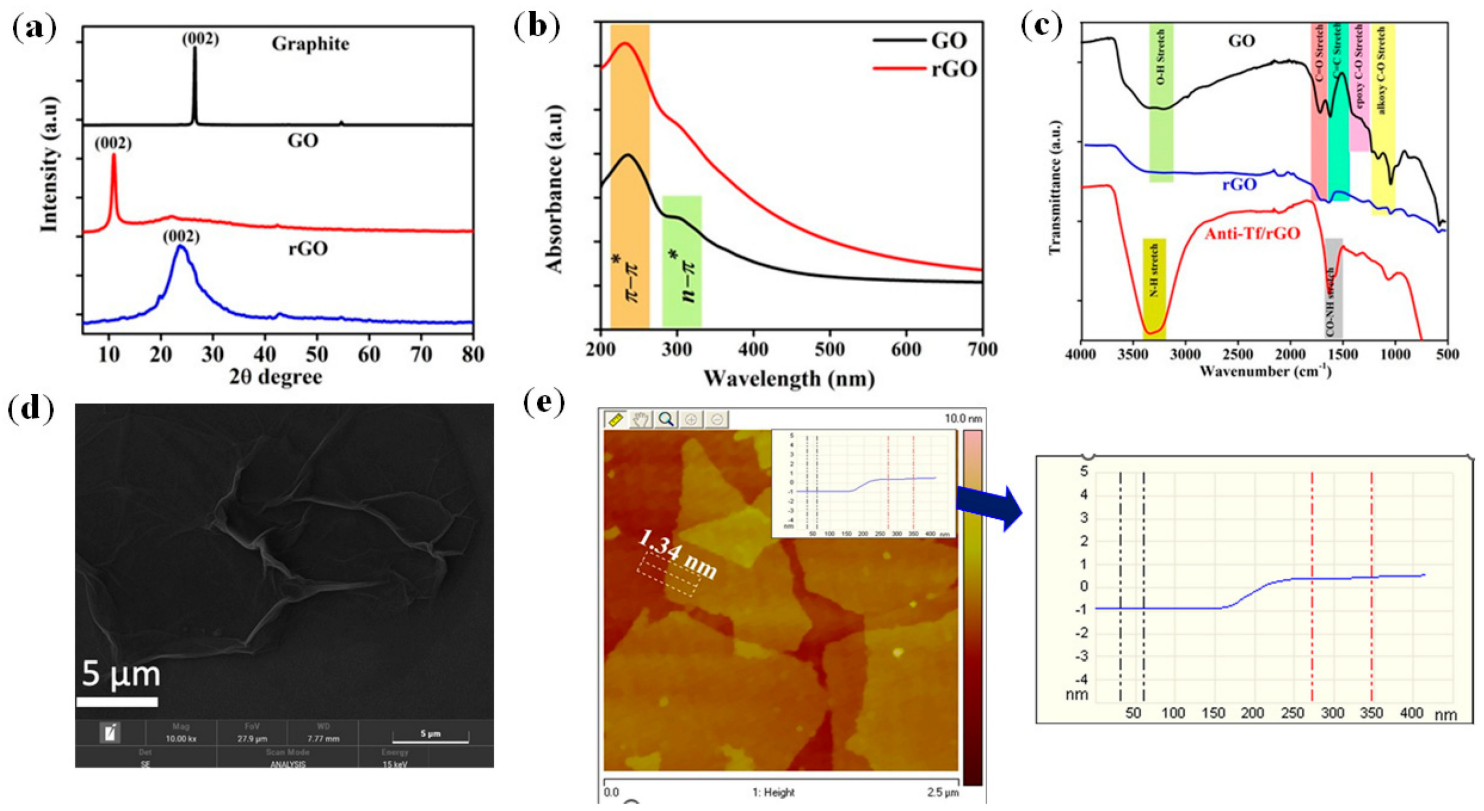
(**a**) X−ray diffraction pattern of GO, graphite, and rGO; (**b**) UV−Visible spectra of prepared GO and rGO; (**c**) FTIR spectroscopy of GO, anti−Tf/rGO, and rGO; (**d**) SEM image of GO; (**e**) AFM image of GO.

Figure 1a shows the XRD diffraction pattern for pristine graphite, GO, and rGO collected in the 2θ range of 5–80°. For pristine graphite, a diffraction peak was observed at 2θ = 26.5°, corresponding to the (002) plane, which agrees with JCPDS file #41-1487. The interplanar spacing of 3.36 Å and full-width half maxima (FWHM) of 0.29 nm, corresponding to the (002) diffraction plane, indicate highly stacked graphitic sheets of the sp^2^ carbon network [47]. Furthermore, the diffraction pattern for GO was observed at 2θ = 10.9°, with a d-spacing of 8.11 Å and FWHM of 0.84 nm, corresponding to the (002) plane. The shift in peaks toward the lower 2θ value and the increase in the interplanar spacing of GO could be attributed to the oxidation and exfoliation of the graphite sheets [48]. Furthermore, the XRD of the rGO exhibited a broad diffraction pattern at around 25°, indicating the reduction of GO and restoration of the graphitic network [49].

Figure 1b shows the UV-Vis absorption spectra of GO. Two absorption peaks at 236 nm and 300 nm were observed and assigned to the π-π* (C=C) and n-π* (C=O) electronic transitions in the GO sheets. This indicates the formation of GO nanosheets [50]. The disappearance of the C=O peak in the UV-Vis spectra of rGO signifies the reduction of GO to rGO. Furthermore, an FTIR study was conducted on the GO nanosheets to recognize the oxygen-containing functional groups, as shown in Figure 1c. The broad peak at 3200–3600 cm^−1^ corresponded to -OH stretching, whereas the peaks at 1714 cm^−1^, 1625 cm^−1^, 1231 cm^−1,^ and 1045 cm^−1^ corresponded to the C=O, C=C, and C-O stretching of the epoxy group and the C-O stretching of the alkoxy group, confirming the structure of the GO nanosheets. CHO functional groups were also present at around 1300 cm^−1^, overlapping with the C-O stretching of the epoxy group [51]. The FTIR spectrum for the rGO film exhibited a reduced number of oxygen-containing functional groups, except for –COOH. The IR bands observed in the region of 3600–2900 cm^−1^ were due to the stretching vibrations of -NH, and the IR band at 1644 cm^−1^ was due to the C=O of the amide bond related to the antibody, indicating the immobilization of anti-Tf antibodies on the surface of the rGO. 

The GO films’ topological and morphological aspects were analyzed using AFM and SEM. As shown in Figure 1d, the SEM micrographs showed folds of crumpled thin sheets, indicating the presence of GO nanosheets. The AFM image (Figure 1e) of the GO nanosheets indicated the formation of single or bilayers of GO with a thickness of 1.34 ± 0.2 nm. 

### 3.2. Electrochemical Characterization of the Fabricated Immunosensor

**Figure 2:**
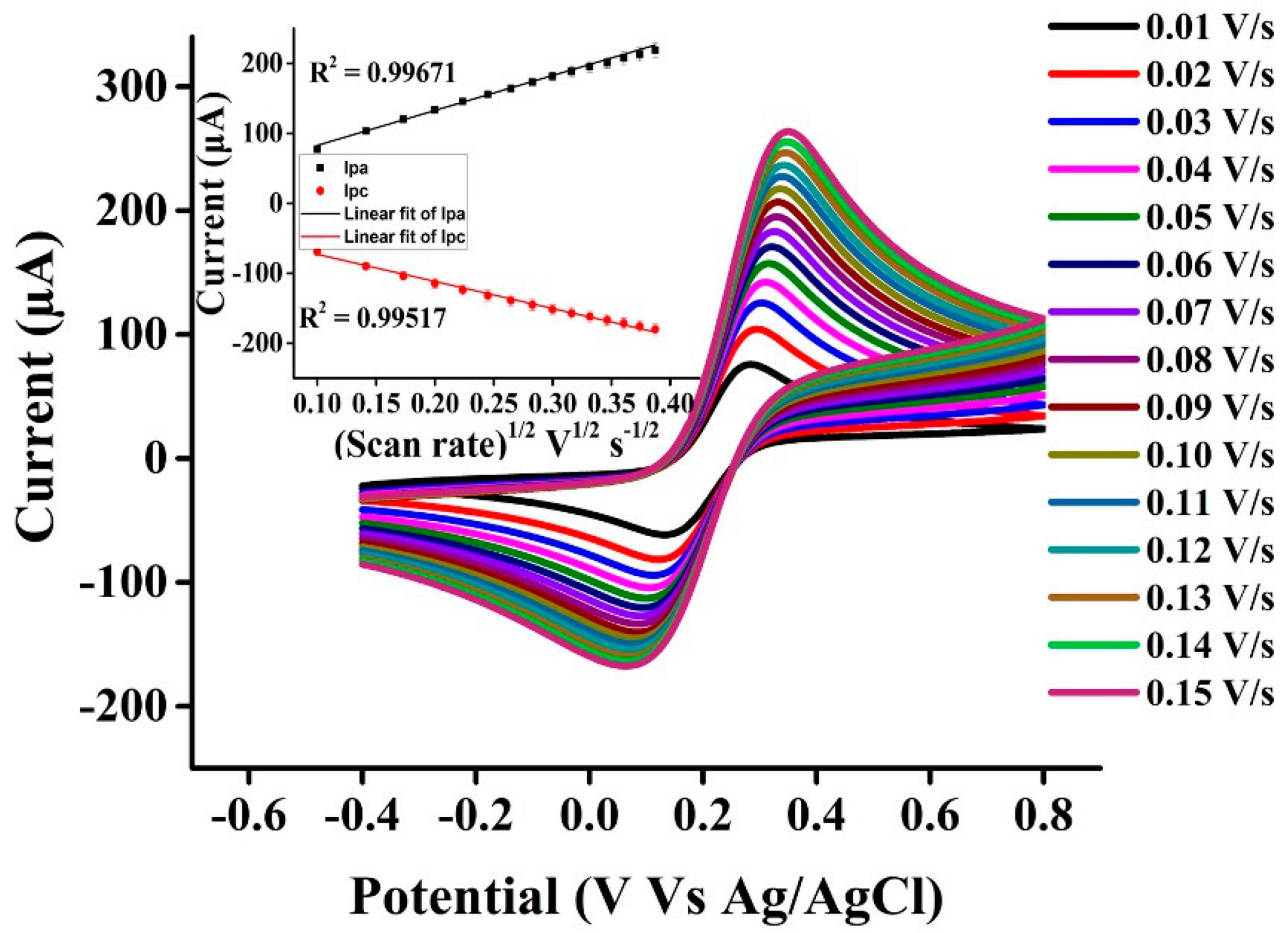
Plot depicting the scan rate range of 0.01 Vs^−1^ to 0.15 Vs^−1^. The inset depicts the linear curve between the square root of the scan rate and the current.

The electrochemical performance of the fabricated rGO/ITO electrodes, comprising redox processes, heterogeneous electron transfer, and adsorption processes of electroactive species, was evaluated using CV output in a 3 mM Zobell’s solution (*n* = 3). The CV curves for the rGO/ITO shown in Figure 2 were recorded at varying scan rates ranging from 0.01 Vs^−1^ to 0.15 Vs^−1^. The results showed an increase in the oxidation peak current (*Ipa)* and a decrease in the reduction peak current (*Ipc*) with increasing scan rates. The change in the cathodic and anodic peak potential separation (*ΔEp*) with the varying scan rates suggests the reversibility/irreversibility of an electrochemical process. The *ΔEp* had a constant value at all scan rates in the case of reversible processes, whereas it increased for irreversible processes. The greater *ΔEp* values and broader redox peaks observed for the rGO/ITO indicated a slower diffusion rate, dominated by mass transfer rather than charge transfer at the electrode surface [52]. In Figure 2, a minor change in the *ΔEp* value with the scan rate can be seen, indicating that the system was slightly irreversible. Moreover, the ratio of the forward and backward peak currents (*Ipa/Ipc*) in the range of 1.132–1.228 indicated the quasi-reversible (1 (quasi-reversible) < *Ipa/Ipc* < 1.27 (reversible)) electrochemical nature at the electrode surface, corroborating the observed variation in the *ΔEp* values at different scan rates. In addition, the inset shown in Figure 2 depicts the linearity between the redox peak currents and the square root of the scan rate, with regression coefficient (R^2^) values of 0.9967 and 0.9951 for *Ipa* and *Ipc,* as described by Equations (2) and (3), indicating the diffusion-limited electrochemical processes at the electrode surface.

*Ipa* (rGO/ITO) *=* 499.74 [Scan rate]^1/2^ + 32.74; R^2^ = 0.9967 (2)

*Ipc* (rGO/ITO) *=* −387.10 [Scan rate]^1/2^ − 34.31; R^2^ = 0.9951; (3)

Furthermore, the diffusion coefficient associated with the redox processes at the rGO/ITO electrode was calculated to be 6.13 × 10^−6^ cm^2^/s using the Randles–Sevcik equation in Equation (4) [53].

(4) $$Ip=\left(2.69\times 10^{5}\right)n^{\frac{3}{2}}\;ACD^{\frac{1}{2}}\;v^{\frac{1}{2}}$$

where *Ip* is the anodic/cathodic peak current in amperes, *n* represents the no. of electrons involved in the redox process (i.e., 1), *C* is the molar concentration of electroactive species (3 × 10^−6^ mol cm^−3^ in the present study), *A* is the electrode area (i.e., 0.25 cm^2^), *ν* denotes the scan rate in Vs^−1^, and *D* represents the diffusion coefficient in cm^2^ s^−1^. The obtained diffusion coefficient of 6.13 × 10^−6^ cm^2^/s indicates a fairly good electron transfer rate. 

**Figure 3:**
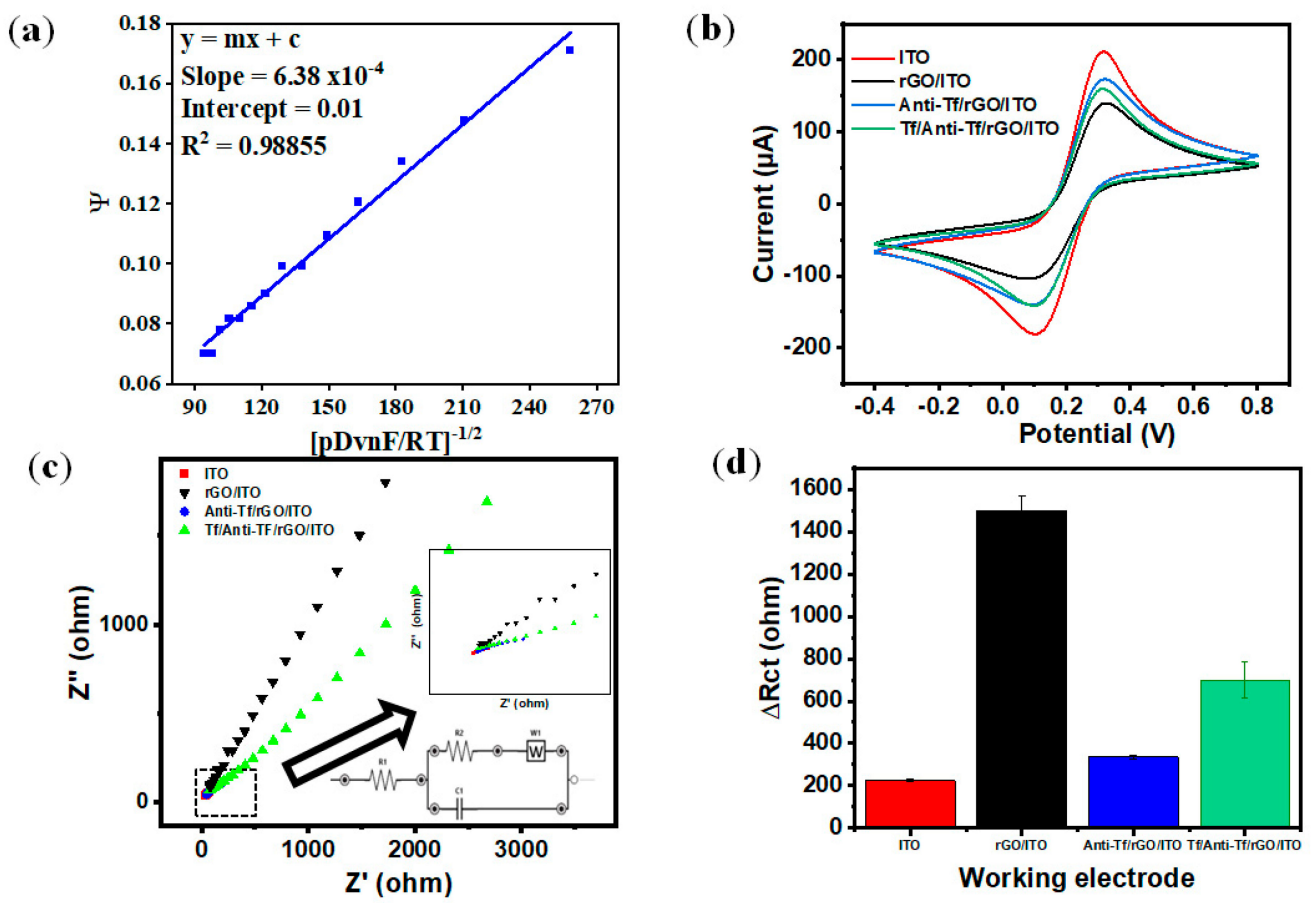
(**a**) Plot of Ψ vs. square root of ΠDνnF/RT for rGO/ITO; (**b**) CV of all modified electrodes; (**c**) EIS response of all modified surfaces with the equivalent circuit model; inset shows a zoomed−in view of all responses; (**d**) comparative histogram of the Rct values of all electrode surfaces, highlighting the increased resistance of the final surface caused by insulation on the electrode surface.

The quasi-reversible behavior and electron transfer kinetics at the rGO/ITO electrode were further verified through the standard heterogeneous electron-transfer rate constant, *K^0^,* which was evaluated using Nicholson’s method in the following Equation (5) [54]:

(5) $$\psi =K^{o}\;\sqrt{\frac{DvnF}{RT}}$$

where *ψ* is Nicholson’s dimensionless kinetic parameter (*ψ* indicates the electrochemical reversibility—when *ψ* = 20, the system is reversible; when *ψ* ≤ 7, the system is quasi-reversible) [55], *D* is the diffusion coefficient in cm^2^ s^−1^, *ν* denotes the scan rate in Vs^−1^, *F* is the Faraday constant, *n* represents the no. of electrons involved, *T* is the temperature (K), and *R* is the universal gas constant. The value of *ψ* was calculated using the Lavagnini et al. method in Equations (6) and (7) [56]:

(6) $$\psi =\frac{\left(-0.6288+0.0021\Delta E_{p}n\right)}{\left(1-0.017\;\Delta E_{p}n\right)}\;$$

(7) $$\psi =2.18\sqrt{\frac{\beta }{\pi }}\;exp[-\left(\frac{\beta^{2}F}{RT}\right)\Delta E_{p}n]$$

where *∆Ep* is the difference in the anodic and cathodic peak potential of <200 mV and >200 mV (for Equations (6) and (7)), and *β* is the transfer coefficient of 0.5. The obtained slope of the linear curve is equal to the *K^0^,* which is 6.38 × 10^−4^ cm s^−1^ and *ψ* = 0.16, confirming the system to be quasi-reversible, as shown in Figure 3a [56].

The obtained value (6.38 × 10^−4^ cm s^−1^) of *K^0^*suggests a quasi-reversible electron transfer process (*K^0^* ≥ 2 × 10^−5^ cm s^−1^), which is in good agreement with the literature [57]. The efficient redox processes at the rGO/ITO surface contribute to the enhanced electron transfer, which can be attributed to the inherent electron transfer property of rGO and, therefore, an increase in the *K^0^* and diffusion coefficient values. Furthermore, the uniformly distributed rGO nanosheets, oxygen-containing groups, and rapid electron transfer through the π-π carbon network and supra-molecular interactions may have contributed to the increased *K^0^* and *D* [58].

The optimized rGO/ITO electrodes were covalently immobilized with anti-Tf using EDC-NHS chemistry to fabricate the biosensing electrodes to detect Tf in the standard and serum samples. The ITO, rGO/ITO, anti-Tf/rGO/ITO, and Tf/anti-Tf/rGO/ITO electrodes were electrochemically characterized stepwise in 3 mM Zobell’s solution using the CV technique at 0.05 Vs^−1^ and are shown in Figure 3b. The CV curves reveal that the oxidation peak current for the rGO/ITO electrode was slightly less than that of the bare ITO electrode and could be attributed to the presence of structural defects in the rGO [59]. These structural defects were expected to impede the electron transfer to the electrodes. Furthermore, the oxidation peak current increased for the anti-Tf/rGO/ITO electrode compared to the rGO/ITO electrode, which may be associated with the electrostatic interaction of (Fe(CN)_6_)^3−/4−^ with the positively charged amine group on the antibody, leading to enhanced electron transport [60]. The increase in the redox currents indicates the successful immobilization of anti-Tf on the rGO/ITO electrode surface. A similar alteration in the current responses after antibody immobilization has been previously reported [25,60,61,62]. Furthermore, the CV curve for the Tf/anti-Tf/rGO/ITO electrode showed a decrease in the redox current, indicating the sluggish electron transfer kinetics due to the antigen–antibody interaction that formed an insulating layer on the electrode surface, restricting the electron transfer [25]. In order to validate the CV results, an EIS experiment was performed (Figure 3c). The Rct values of the ITO, rGO/ITO, anti-Tf/rGO/ITO, and Tf/anti-Tf/rGO/ITO electrodes were 224.7 (±0.3) Ω, 1500.2 (±0.5) Ω, 335.5 (±0.2) Ω, and 700.2 (±0.1) Ω, respectively, as shown in Figure 3d. The EIS data validated the CV results and confirmed the successful fabrication of antibody/antigen conjugates.

### 3.3. Detection of Tf Antigen Using Fabricated Immunosensor

**Figure 4:**
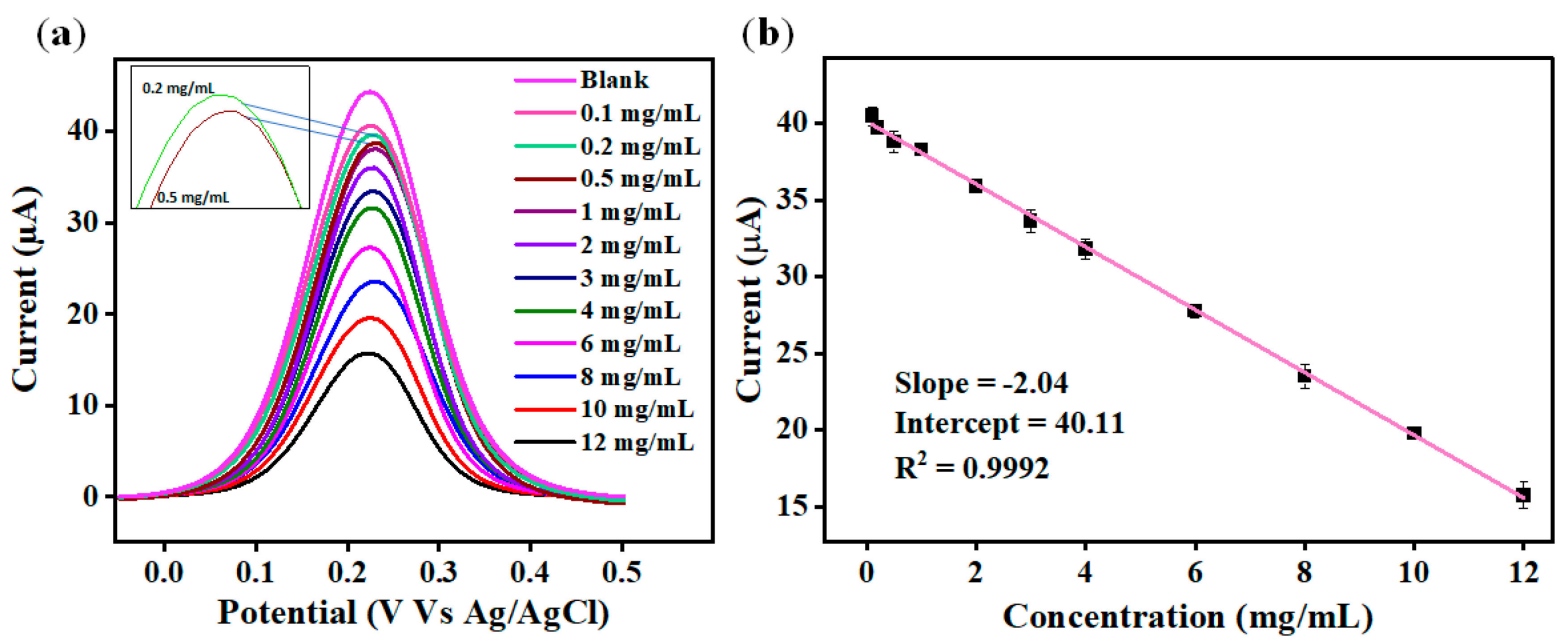
(**a**) DPV curves depicting the responses of the anti−Tf/rGO/ITO immunosensor to different concentrations of Tf; (**b**) calibration curve showing the relationship between the response current of the immunosensor and the varying concentrations of Tf antigen.

A more sensitive electrochemical technique, i.e., DPV, which only provides the oxidation peak corresponding to the Faradic current, was used to analyze the detection performance of the anti-Tf/rGO/ITO electrode in 3 mM Zobell’s solution. The anti-Tf/rGO/ITO electrodes were exposed to varying concentrations of antigens ranging from 0.1 mg mL^−1^ to 12 mg mL^−1^, and the DPV responses were recorded for each concentration, as shown in Figure 4a. The responses of each concentration of the Tf/anti-Tf/rGO/ITO electrodes were analyzed in triplicate. With a rise in the antigen concentration, a decrease in the peak current was observed. The decreasing trend in the current can be attributed to the increasing antibody–antigen interaction that formed a non-conductive layer, resulting in hindered electron transfer.

The designed immunosensor exhibited linearity in the range of 0.1 mg mL^−1^ to 12 mg mL^−1,^ as shown in Figure 4b. The regression coefficient of the current vs. concentration curve was obtained using Equation (8) and was estimated to be R^2^ = 0.9992.

*Ip*(μA) = −2.04 [Tf concentration in mg mL^−1^] + 40.11; R^2^ = 0.9992 (8)

Furthermore, the sensitivity of the anti-Tf/rGO/ITO immunosensor electrode was determined to be 2.04 ± 0.01 mg mL^−1^ from the slope of the concentration linear response curve. The LOD and the limit of quantification (LOQ) were estimated using Equations (9) and (10), respectively:

(9) $$LOD=k\;\;\frac{\sigma }{S}$$

(10) $$LOQ=k_{Q}\;\frac{\sigma }{S}$$

where, *k* denotes a statistical confidence parameter (*k* = 3.3), *σ* represents the standard deviation of the blank, *S* denotes the electrode’s sensitivity obtained from the slope of the calibration curve, and *k_Q_* = 10 is also a statistical confidence parameter.

The LOD and LOQ of the fabricated immunosensor were estimated to be 0.010 ± 0.007 mg mL^−1^ and 0.034 ± 0.007 mg mL^−1^, respectively.

### 3.4. Specificity and Stability of the Immunosensor

**Figure 5:**
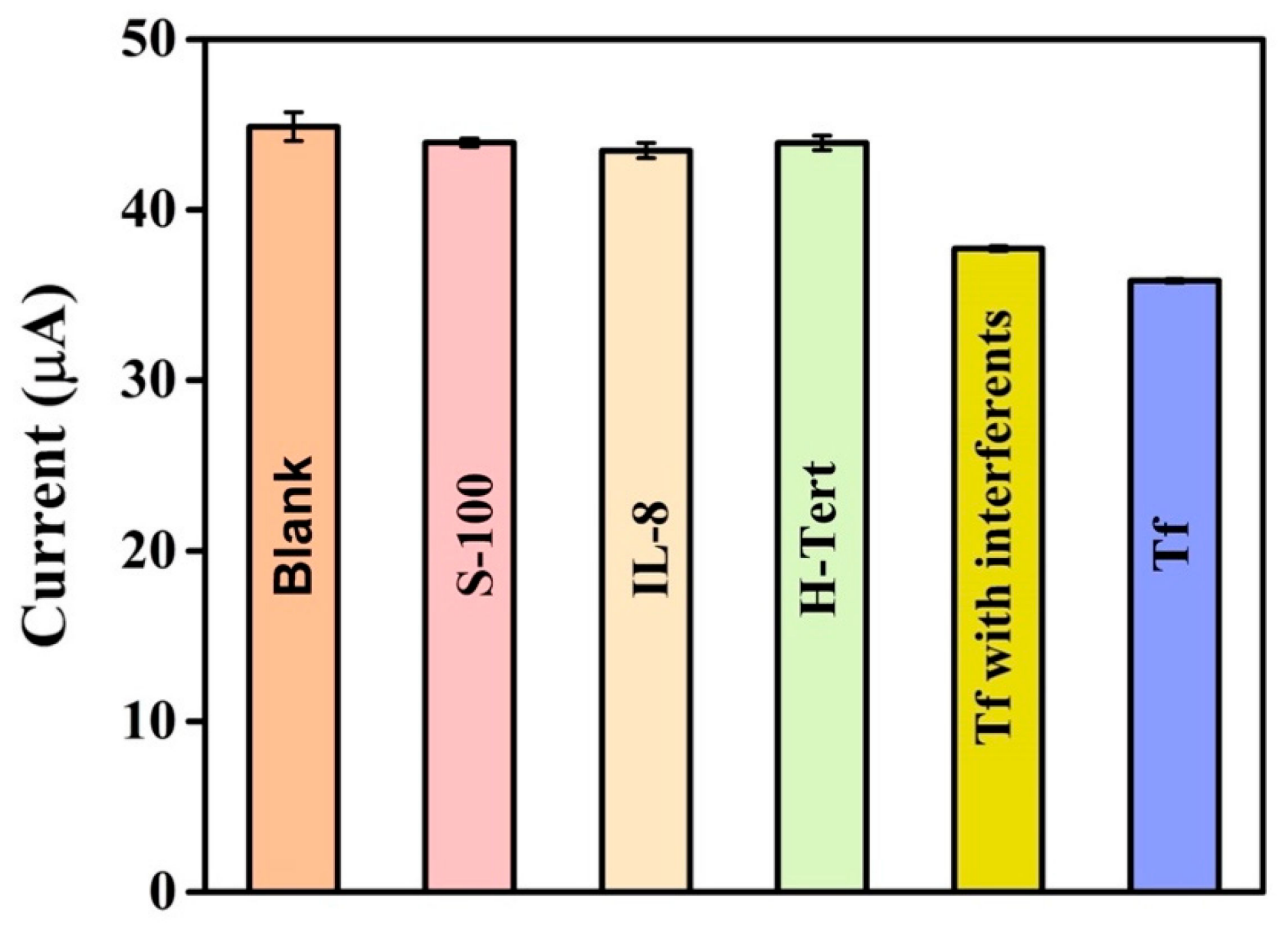
Specificity analysis of the immunosensor showing the high specificity of the immunosensors toward the Tf antigen in the presence of different interferents. Tf with interferents includes a mixture of all possible interferents and Tf.

Specificity and stability are critical parameters for any biosensor to ensure accurate and reliable results. The specificity of the fabricated immunosensor was evaluated by exposing it to various nonspecific antigens such as S-100, IL-8, h-TERT, and a mixture of these with Tf, each with a concentration of 2 mg mL^−1^ (n = 3). Figure 5 shows the current response of the nonspecific antigens, along with the blank and Tf antigens. It can be seen that the responses of all the nonspecific antigens were equivalent to the response signal of the blank, indicating the significant specificity of the immunosensors toward the target antigen due to the non-binding of the nonspecific analyte to the bio-recognition sites. Furthermore, a significant decrease in the current response of the fabricated immunosensor was observed on exposing it to the sample containing a mixture of all analytes and a specific Tf antigen, confirming the high specificity toward Tf detection in mixed samples. The high sensitivity and specificity exhibited by the immunosensor indicate its potential for clinical applications. 

The proposed immunosensor was stored at 4 °C and DPV responses were recorded every month for a Tf concentration of 1 mg mL^−1^. The current responses of the immunosensor remained stable, with a signal retention of 95.89% for up to 4 months, demonstrating significant binding between Tf and the antibody, even after several months. After 7 months, the signal retention decreased to 90.32%, indicating that the electrode remained highly stable for a reasonable amount of time.

### 3.5. Validation of Immunosensor Results Using ELISA and Autobioanalyzer

**Figure 6:**
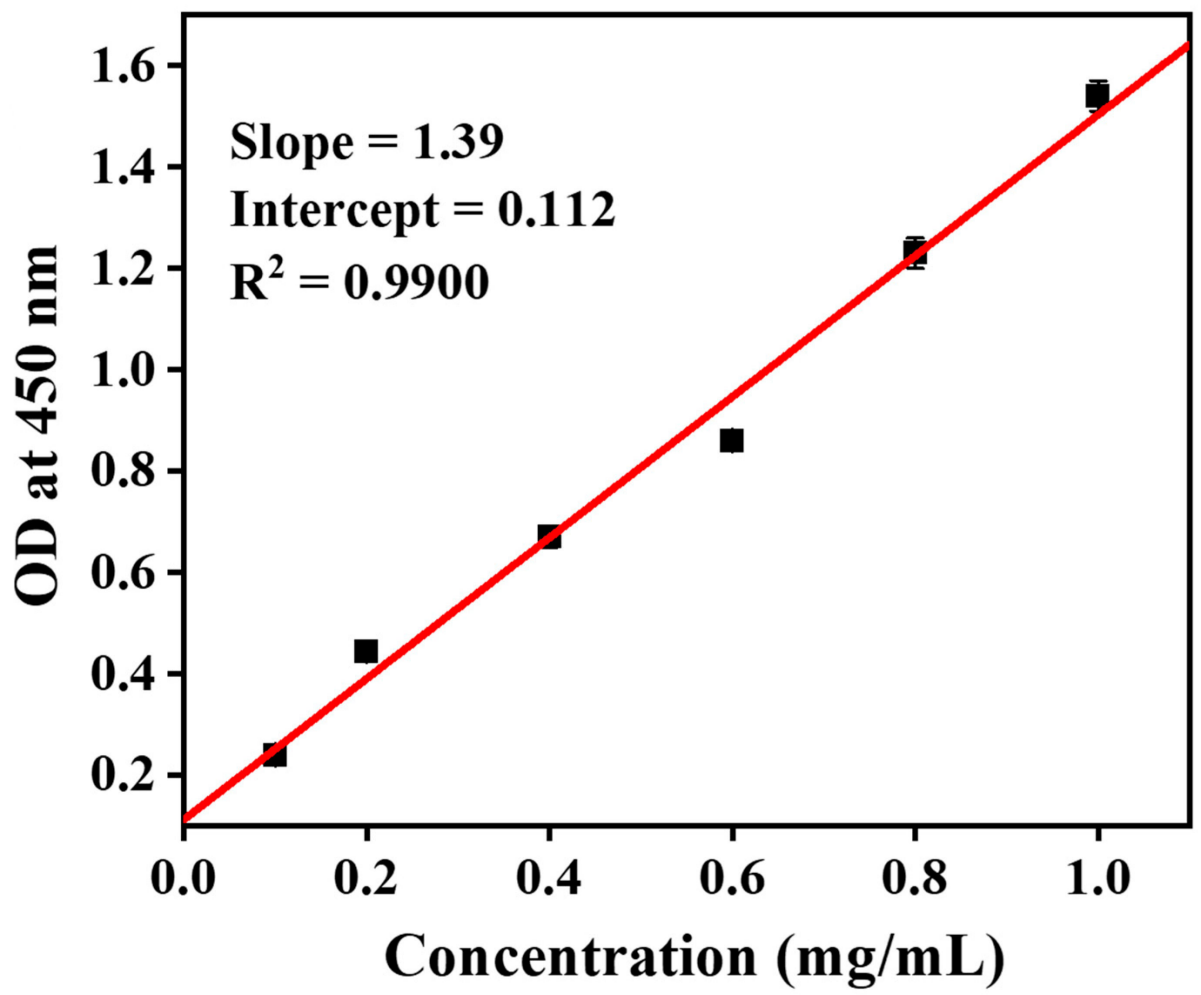
Standard curve of serum Tf levels estimated using ELISA.

ELISA was performed to validate the Tf levels obtained from the fabricated immunosensor. Briefly, a Tf-coated ELISA plate was exposed to undiluted (20 μL) serum samples from different cancer patients, and the absorbance was measured at 450 nm (n = 3). A standard graph, shown in Figure 6, was plotted to calculate the unknown concentrations of serum Tf levels of the various cancer patients. The ELISA results demonstrated a significant decrease in the serum Tf levels of the various cancer patients compared to the standard values provided by the National Institute of Health (NIH) [63]. We observed that the serum Tf levels were between 0.049 and 0.329 mg mL^−1^ among the various cancer patients of the chosen group.

Serum Tf levels were also calculated by estimating the serum ferritin levels using an autobioanalyzer, which was clinically approved. The literature suggests that 1 mg of Tf could bind to 1.4 µg of iron; based on this, the serum Tf levels were calculated from the obtained ferritin levels. 

### 3.6. Validating the Immunosensor with Real Samples

**Figure 7:**
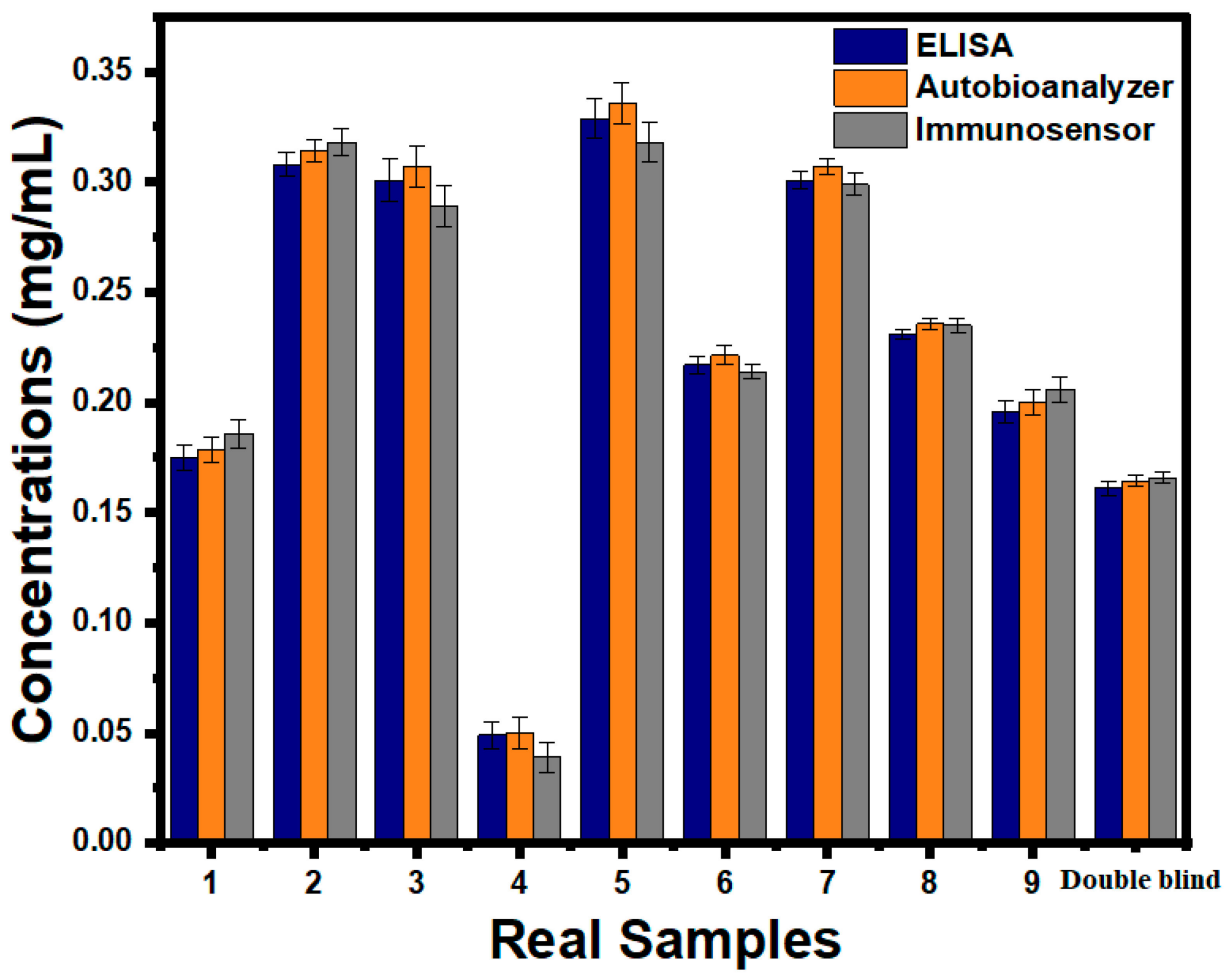
Comparative graph showing the results of the serum sample studies using the anti-Tf/rGO/ITO immunosensor, ELISA, and autobioanalyzer.

The field applicability of the fabricated immunosensor was analyzed by examining its performance on real samples. The immunosensor was tested on serum samples collected from 10 patients with different cancers, following the ethical clearance guidelines discussed in Section 2.2. Out of the 10 samples, 1 was double-blinded. The anti-Tf/rGO/ITO immunosensor was exposed to undiluted 20 μL of serum samples from different cancer patients, and the responses were recorded using DPV in Zobell’s solution (*n* = 3). The immunosensor exhibited excellent performance. The concentrations of Tf obtained using the fabricated immunosensor were compared with those of standard techniques such as ELISA and an autobioanalyzer. Figure 7 shows a comparison of the results from the fabricated immunosensor with those of the standard techniques (ELISA and autobioanalyzer), showing the concordance of the immunosensor with the other two routinely used techniques in hospitals. The constructed immunosensor’s remarkable performance for cancer detection was demonstrated by <5% deviation in the observed Tf concentrations.

## 4. Conclusions

We successfully fabricated an anti-Tf/rGO/ITO electrochemical immunosensor for the electrochemical detection of Tf antigen in serum samples of cancer patients. The surface of the fabricated immunosensor was physically characterized using XRD, UV-Vis spectroscopy, FTIR, SEM, and AFM. The stepwise fabrication of the immunosensor was performed electrochemically using CV and EIS. The calibration study was performed using highly sensitive DPV techniques, and the results demonstrated a wide dynamic range from 0.1 mg mL^−1^ to 12 mg mL^−1^, hence it is good enough to detect the clinical range of Tf between 2 and 3.6 mg mL^−1^. The anti-Tf/rGO/ITO immunosensor had a LOD of 0.010 ± 0.007 mg mL^−1^, a LOQ of 0.034 ± 0.007 mg mL^−1^, and a sensitivity of 2.04 ± 0.01 mg mL^−1^. This strongly suggests that the designed sensor is highly sensitive and can precisely measure Tf in the clinical range, highlighting its immense commercial potential. In addition, it exhibits great specificity toward the target antigen and is extremely stable, with a shelf life of up to several months. Furthermore, the performance of the anti-Tf/rGO/ITO for Tf detection was validated by gold-standard ELISA and autobioanalyzer techniques, which demonstrated a variability of < 5%. The results indicate that the fabricated rGO-based immunosensor can serve as a cost-effective, rapid, and selective diagnostic tool for the diagnosis and prognosis of cancer, as well as other diseases where Tf is used as a diagnostic marker.

## Data Availability

Not applicable.
